# Dr. James Henry Etheridge

**Published:** 1899-03

**Authors:** 


Dr. James Henry Etheridge, of Chicago, died at his residence in that city, Thursday, February 9, 1899, in the 55th year of his age. The cause of his death, which was sudden, is reported as fibrous myocarditis, consequent upon coronary sclerosis.

Dr. Etheridge was born at St. Johnsville, N. Y., March 20, 1844, of English parentage. After preliminary preparation in the schools of his vicinity and in his father's office, he entered Rush Medical College, from which he was graduated in 1869. Two years afterward he went to Europe where he spent some time in the hospitals of the old world. In 1871 he reëstablished himself in practice which he continued without interruption until his death. Of late years he devoted himself almost exclusively to gynecic surgery, and occupied the chair of gynecology at Rush College, with which of late obstetrics had been united.

Dr. Etheridge was one of the hard workers in the profession. He was a rapid and deft operator, a man of excellent judgment and commanded the universal respect of the profession and the public. He is survived by a widow and two daughters.

